# Localization of dendritic cells and T cells within the tumor microenvironment in different types of skin cancer

**DOI:** 10.1007/s00418-026-02468-8

**Published:** 2026-04-25

**Authors:** Marina Wanner, Anna Brunner, Daniela Ortner, Christoph H. Tripp, Martin Hermann, Selina Neurauter, Barbara Del-Frari, Van Anh Nguyen, Patrizia Stoitzner

**Affiliations:** 1 Department of Dermatology, Venereology, and Allergology, Medical University of Innsbruck, Anichstraße 35, 6020 Innsbruck, Austria; 2 Department of Anesthesia and Intensive Care Medicine, Medical University Innsbruck, Innsbruck, Austria; 3 Department of Plastic, Reconstructive and Aesthetic Surgery, Medical University Innsbruck, Innsbruck, Austria

**Keywords:** Skin cancer, Tumor microenvironment, Dendritic cells, T cells, Immunophenotyping, Immunotherapy

## Abstract

Immunophenotyping of tumor-infiltrating immune cells is increasingly important for understanding the tumor microenvironment (TME), particularly in the diagnosis and treatment of skin cancer. CD1a^+^ dendritic cells (DC) initiate T cell activation by presenting tumor antigens, while T cells directly target tumor cells. Analyzing their spatial distribution in different types of skin cancer can provide insights into immune response patterns. To characterize the immune cell composition within the TME, we performed immunofluorescence staining for CD1a and CD3 on formalin-fixed, paraffin-embedded (FFPE) samples from actinic keratoses (*n* = 18), squamous cell carcinoma (*n* = 23), basal cell carcinoma (*n* = 19), and melanoma (*n* = 22), with nevi (*n* = 16) and healthy skin (*n* = 9) as controls. Immune cells were quantified across four tumor compartments: intratumoral, tumor margin, intraepidermal, and intradermal. Both CD1a^+^ DC and CD3^+^ T cells were detected across all tumor entities, displaying distinct spatial distribution patterns. DC were enriched intratumorally and within the epidermis, whereas T cells predominantly accumulated at the tumor margin (main effect of region, *p* < 0.001). Melanoma exhibited significantly fewer DC at the tumor margin while maintaining strong T cell infiltration. Overall, the immune architecture of skin tumors is highly compartmentalized, characterized by region-specific DC and T cell distributions. These findings underscore the relevance of spatial immune profiling for understanding immune escape mechanisms and informing immunotherapeutic strategies.

**Supplementary Information:**

The online version contains supplementary material available at 10.1007/s00418-026-02468-8.

## Introduction

The incidence of skin cancer worldwide has steadily increased over recent decades, with epidemiological data demonstrating a significant rise in both melanoma and non-melanoma skin cancers (NMSC) in most regions (Zhou et al. 2025). The skin, the body’s largest organ, serves as a critical barrier against environmental insults, particularly the carcinogenic effects of ultraviolet (UV) radiation (Savoye et al. 2018). Skin cancer encompasses a spectrum of malignancies, ranging from the relatively indolent basal cell carcinoma (BCC) to aggressive melanoma characterized by rapid metastatic spread (Naik 2021; Hasan et al. 2023). Understanding the tumor microenvironment (TME) is essential for developing therapies that improve patient outcomes.

Actinic keratosis (AK) is a precancerous skin lesion that has the potential to progress to squamous cell carcinoma (SCC). AK is therefore a critical target for early detection and intervention (Eisen et al. 2021). The term “actinic” refers to its UV-induced origin, while “keratosis” refers to the abnormal keratinization of the epidermis (Reinehr and Bakos 2019). SCC arises from atypical keratinocytes and is primarily caused by chronic UV exposure (Wysong 2023). BCC is the most common type of skin cancer and, although it rarely metastasizes, it can cause significant morbidity due to local invasion and tissue destruction (Seidl-Philipp et al. 2021). In contrast, malignant melanoma, although less frequent, is associated with substantially higher mortality due to its aggressive nature and propensity for early metastasis (Garbe et al. 2022b; Joshi et al. 2025).

Surgical excision remains the primary treatment for non-metastatic skin cancers. Alternative therapies for managing precancerous lesions, such as cryotherapy, photodynamic therapy, and topical treatments, are also available (Leiter et al. 2020; Algarin et al. 2023). For metastatic or unresectable skin tumors, immunotherapy and targeted therapy represent the current standard of care (Peris et al. 2023; Garbe et al. 2022a; Kreidieh and Wong 2025). Immune checkpoint inhibitors (ICIs) have significantly increased the 5-year overall survival rate in patients with metastatic melanoma (Wolchok et al. 2025). However, intratumoral heterogeneity, whereby tumors exhibit genetic and phenotypic diversity, is a well-established mechanism by which they evade immune surveillance and potentially undermine the efficacy of immunotherapies. Understanding the immune cell distribution within the TME could provide valuable insights for improving therapeutic outcomes (Roerden and Spranger 2025).

The aim of this study is to improve our understanding of the TME in skin cancer by investigating the presence and spatial distribution of key immune cell types, namely CD1a^+^ dendritic cells (DC) and CD3^+^ T cells. DC are potent antigen-presenting cells that are essential for initiating and maintaining both primary and secondary immune responses (Steinman and Hemmi 2006). Among these, Langerhans cells (LC), a specialized subset of DC, are predominantly located in the suprabasal layer of the epidermis and are characterized by their unique Birbeck granules. LC express both Langerin and CD1a, which are key markers for identifying them (Romani et al. 2010). Dermal DC also express CD1a, along with a variety of other markers that help distinguish different DC subtypes, such as conventional type 1 DC (cDC1) and conventional type 2 DC (cDC2). This highlights the phenotypic and functional diversity within this cell population (Clausen and Stoitzner 2015).

T cells are essential to adaptive immunity and are characterized by their expression of CD3. They consist of various subsets, including CD4^+^ helper T cells, particularly those of the Th1 type. These cells recognize peptides presented on MHC class II molecules on DC to coordinate immune responses, while CD8^+^ cytotoxic T cells engage MHC class I peptide complexes to directly target and eliminate tumor cells (Bawden et al. 2024). Additionally, regulatory T cells, a specialized subset of CD4^+^ T cells, play a crucial role in maintaining immune homeostasis by suppressing excessive immune responses (Tay et al. 2023).

Integrating immunophenotyping into both research and clinical practice has transformed our diagnostic and prognostic approach to skin cancer (Voiculescu et al. 2025). Profiling tumor-infiltrating immune cells provides valuable insights into the TME, which is a critical factor in cancer initiation, progression, and treatment resistance (Lopez de Rodas et al. 2025; Bida et al. 2024). This shift in perspective has changed our understanding of cancer, viewing it not simply as a mass of malignant cells but as a dynamic interplay between the tumor and the immune system (Ravi et al. 2025).

We used immunofluorescence (IF) staining to map the spatial distribution of DC and T cells within distinct tumor regions across different human skin cancer types (AK, SCC, BCC, and melanoma), providing valuable insights into the TME.

## Materials and methods

### Study population

Formalin-fixed, paraffin-embedded (FFPE) tissue blocks from patients diagnosed with various skin cancers (AK, SCC, BCC, and primary melanoma) were obtained from the Biobank of the Department of Dermatology, Venereology, and Allergology at the Medical University of Innsbruck, Austria. Written informed consent was obtained from all participants prior to inclusion in the study. The study protocol was approved by the Ethics Committee of the Medical University of Innsbruck (EK Nr. 1177/2017, EK Nr. 1170/2019).

Our cohort comprised 82 FFPE tumor specimens, including AK (*n* = 18), SCC (*n* = 23), BCC (*n* = 19), and melanoma samples (*n* = 22). To enable direct comparison of melanoma with its benign counterpart, we also included a set of nevi (*n* = 16). Additionally, the Department of Plastic, Reconstructive, and Aesthetic Surgery provided 9 samples of healthy skin from anonymous donors undergoing breast reduction or abdominoplasty procedures to serve as controls (EK AN5003). Demographic and clinical data were collected, including age, sex, tumor thickness (for melanoma), tumor location, and histological subtype.

### Immunofluorescence staining

Tissue sections, each 3 µm thick, were mounted on Xtra-adhesive slides (Leica, Wetzlar, Germany). A 2-day protocol for antigen retrieval and staining optimization was employed to ensure high signal quality. On the first day, tissue sections were deparaffinized using a Gemini Automated Slide Stainer with 100% xylene (3 × 2 min, uniLAB, Heidelberg, Germany) followed by washes in 100% ethanol (2 × 2 min, uniLAB), 96% ethanol (2 × 2 min, uniLAB), 80% ethanol (1 × 3 min, uniLAB), and deionized water (5 min). Heat-induced antigen retrieval was performed in a microwave oven on a low setting using a sodium citrate buffer (10 mM, pH 6.0, Merck, Darmstadt, Germany) for 4 cycles of 5 min each, with 2-min cooling intervals between each cycle. After cooling for 30 min at room temperature (RT), the slides were rinsed twice with phosphate-buffered saline (PBS; Gibco, Thermo Fisher Scientific, Waltham, MA, USA) for 5 min each. For blocking, the tissue sections were incubated in 10% goat serum (Sigma-Aldrich, St. Louis, MO, USA) in 0.5% Triton X-100 (Sigma-Aldrich)/PBS (Gibco) for 1 h at RT in a humidified chamber. Primary monoclonal antibodies against CD1a (clone O10, 1:100; Novus Biologicals, Littleton, CO, USA) and CD3 (clone OKT3, 1:100; BioLegend, San Diego, CA, USA) were diluted in 1% bovine serum albumin (BSA, Serva, Heidelberg, Germany)/PBS and applied to each section, followed by incubation overnight at 4 °C in a humidified chamber.

On day 2, after washing in PBS 3 times for 5 min each, sections were incubated with the appropriate secondary antibodies, anti-mouse IgG_2_a-Alexa Fluor 594 (1:2000; Invitrogen, Thermo Fisher Scientific) for CD3 and anti-mouse IgG_1_-Alexa Fluor 488 (1:1000; Invitrogen) for CD1a, prepared in 1% BSA/PBS. The slides were then incubated for 90 min at RT in a humidified chamber. This step was followed by three washes in PBS for 5 min. Finally, the tissue sections were mounted with Vectashield® Antifade mounting medium containing 1.5 µg/mL of 4′,6-diamidino-2-phenylindole (DAPI, Vector Laboratories, Burlingame, CA, USA) for nuclear staining. The slides were stored at 4 °C until further analysis.

### Cell counting and analysis

Regions of interest within the tissue sections were classified into four areas: intratumoral/intralesional, tumor margin/lesion margin, intraepidermal, and intradermal. In healthy skin samples, epidermis and dermis were distinguished. A fluorescence microscope (Olympus BX60, Tokyo, Japan, filter cube UM61002 D/F/TXRD 506) was used to visualize CD1a^+^ DC and CD3^+^ T cells. Five to eight representative images per region (field of view) were captured at ×400 magnification using the Gryphax software (Jenoptik, Jena, Germany). CD1a^+^ DC and CD3^+^ T cells were identified by green (Alexa Fluor 488) and red (Alexa Fluor 594) fluorescence, respectively. ImageJ (version 1.53k, US National Institutes of Health, Bethesda, MD, USA) was used to quantify the number of CD1a^+^ DC and CD3^+^ T cells in these areas. The average cell count, derived from five to eight images per area, was analyzed using Microsoft Excel (Microsoft, Redmond, WA, USA).

### Statistical analysis

Statistical analysis was performed using IBM SPSS Statistics (version 27.0.1, IBM Corp., Armonk, NY, USA). Mean values and standard deviations (SD) were calculated from ImageJ-based counts. Group differences were assessed using a multivariate analysis of variance (MANOVA) with region and tumor entity as fixed factors, followed by univariate ANOVA and pairwise post hoc comparisons using Bonferroni correction; effect sizes reported as partial (η^2^). Associations between CD1a^+^ DC and CD3^+^ T cells were analyzed using Spearman’s correlation coefficients. IF and IHC were compared using mixed-effects models accounting for paired tumor specimens and region-specific effects. Graphs were generated using GraphPad Prism (version 5.0; GraphPad Software, San Diego, CA, USA). A *p* value < 0.05 was considered statistically significant.

## Results

The study included 107 skin specimens from 98 patients (42 female, 56 male) with skin tumors or nevi, and 9 anonymous donors who provided healthy skin samples as controls. Tumor specimens included AK (*n* = 18), SCC (*n* = 23), BCC (*n* = 19), and primary melanoma (*n* = 22). To compare malignant and benign melanocytic lesions, an additional set of nevi (*n* = 16) was analyzed. The mean patient age was 61.4 years (range 19–92 years), and the head and neck region was the most frequently affected site (41% of cases). Detailed demographic and clinicopathological data, including tumor thickness (Breslow depth for melanoma) and histological subtypes, are summarized in Supplementary Tables 1–5.

**Fig. 1:**
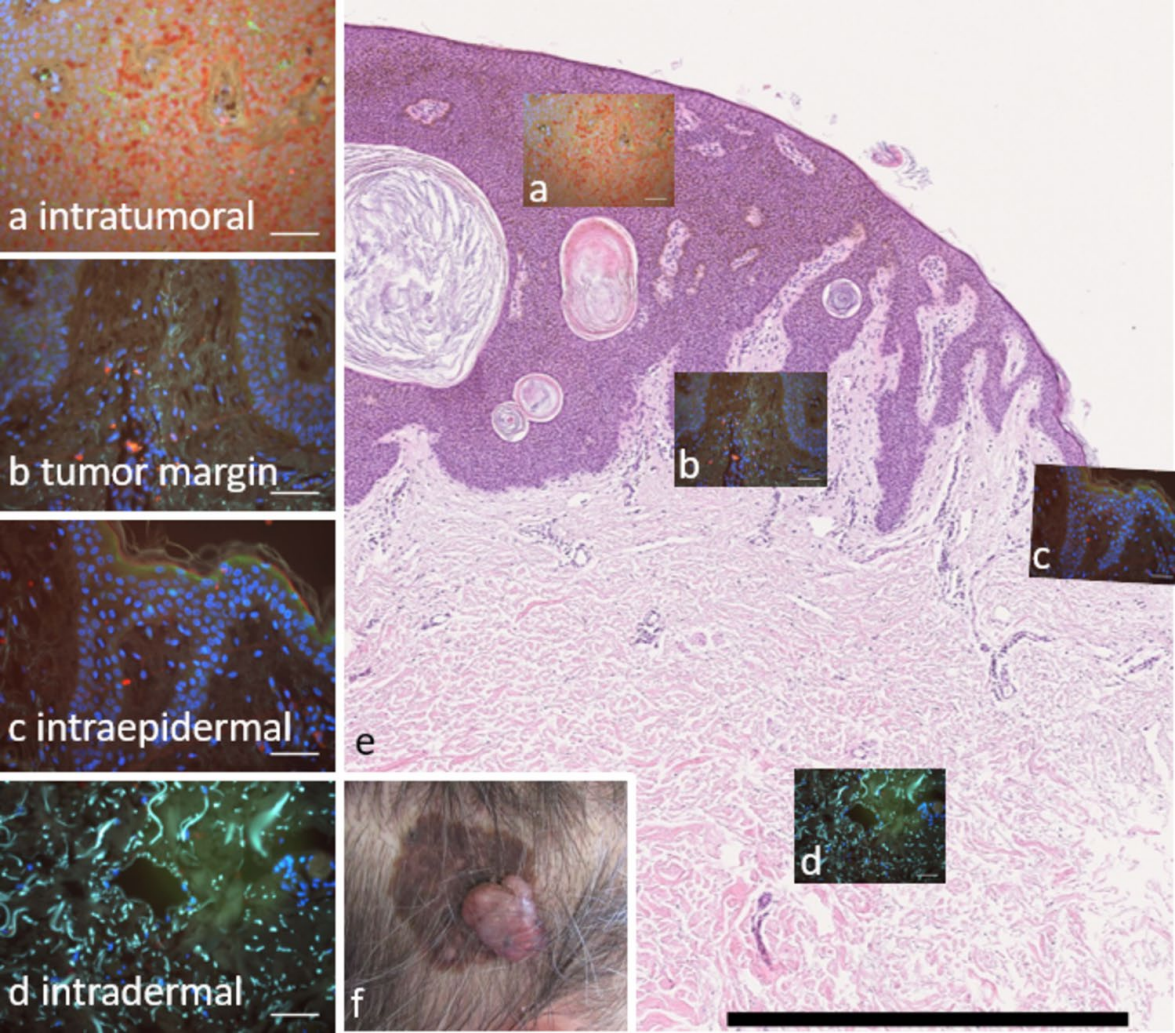
Spatial overview of a melanoma sample with region-specific IF analysis. Representative IF images of thefour analyzed compartments are shown: (a) intratumoral, (b) tumor margin, (c) intraepidermal, and (d) intradermal.The locations of the immunofluorescence images are indicated within an overview histology section of the samemelanoma sample. (e) Hematoxylin and eosin–stained overview section and (f) corresponding clinical presentation. Scale bars, 50 μm (all IF images) and 1000 μm (histology)

To analyze the distribution of CD1a^+^ DC and CD3^+^ T cells, four compartments were defined for each entity: intratumoral/intralesional, tumor margin/lesion margin, intraepidermal, and intradermal (Fig. 1). For each sample, five to eight representative images per region were acquired using a fluorescence microscope. This approach ensured systematic coverage of each compartment while maintaining consistency across all samples.

In healthy skin, we analyzed the epidermal and dermal compartments to provide a baseline for comparison with cancerous tissue. Fluorescence images were processed using ImageJ software to allow precise quantification of immune cells. CD1a^+^ DC were identified by staining with a secondary antibody conjugated to Alexa Fluor 488 (green fluorescence), while CD3^+^ T cells were visualized with Alexa Fluor 594 (red fluorescence). Average cell counts were calculated for each region based on multiple images and used for statistical comparisons between tumor types and regions.

### CD1a^+^ DC and CD3^+^ T cell staining in skin cancer and healthy controls

**Fig. 2:**
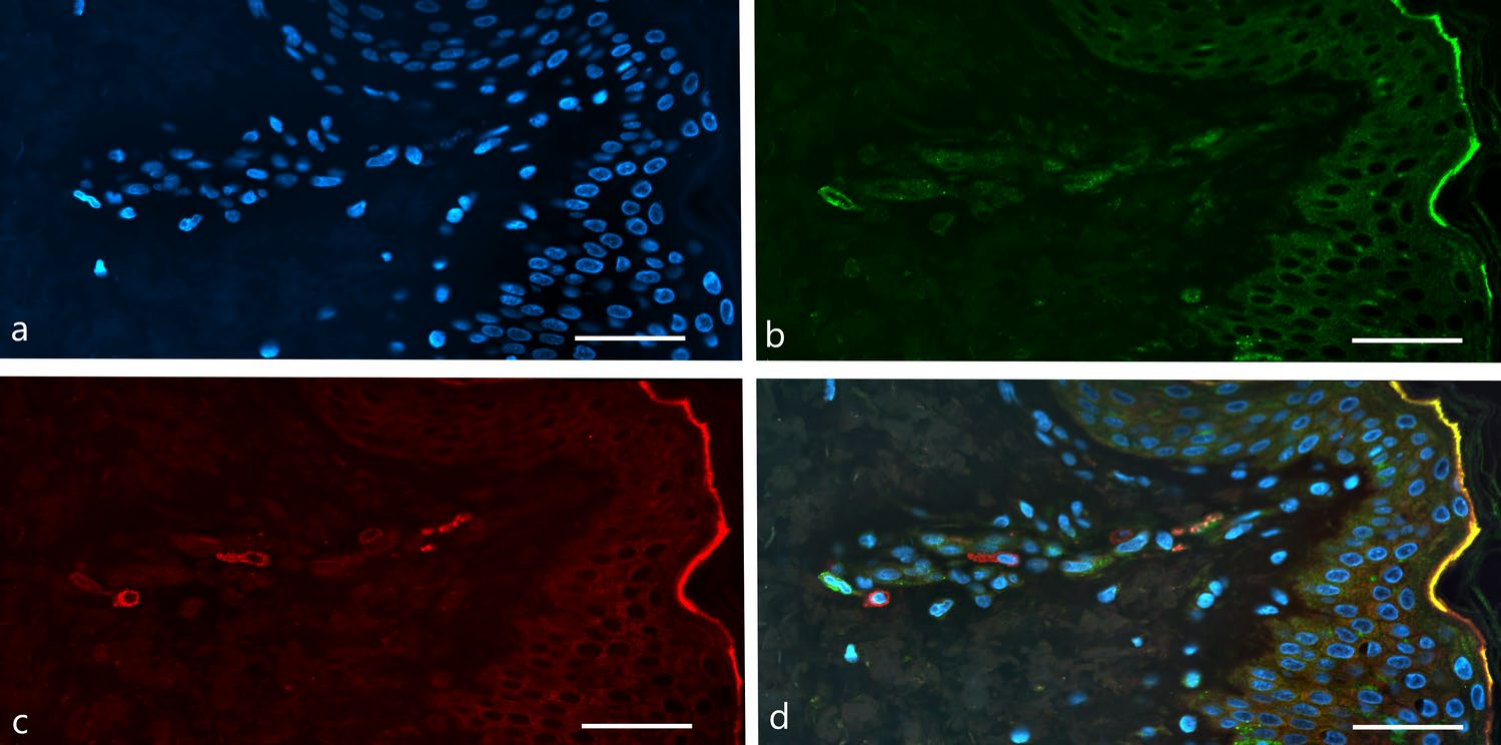
IF staining of healthy adult human skin. Representative images showing nuclear staining with DAPI (blue; a), CD1a⁺ DC (green, Alexa Fluor 488; b), CD3^+^T cells (red, Alexa Fluor 594; c), and the merged image displaying allfl uorochromes (d). Scale bar, 50 μm

To validate the IF staining protocol, we performed staining on healthy control skin. As shown in Fig. 2, in the representative healthy skin sample, CD1a^+^ DC were visualized using Alexa Fluor 488 (green fluorescence) and CD3^+^ T cells using Alexa Fluor 594 (red fluorescence). CD1a^+^ DC were predominantly localized in the epidermis, while CD3^+^ T cells were concentrated in the dermis, close to the epidermal layer. Fluorescence microscopy revealed occasional close proximity between CD1a^+^ DC and CD3^+^ T cells in the merged images, suggesting potential cell-cell interactions.

**Fig. 3:**
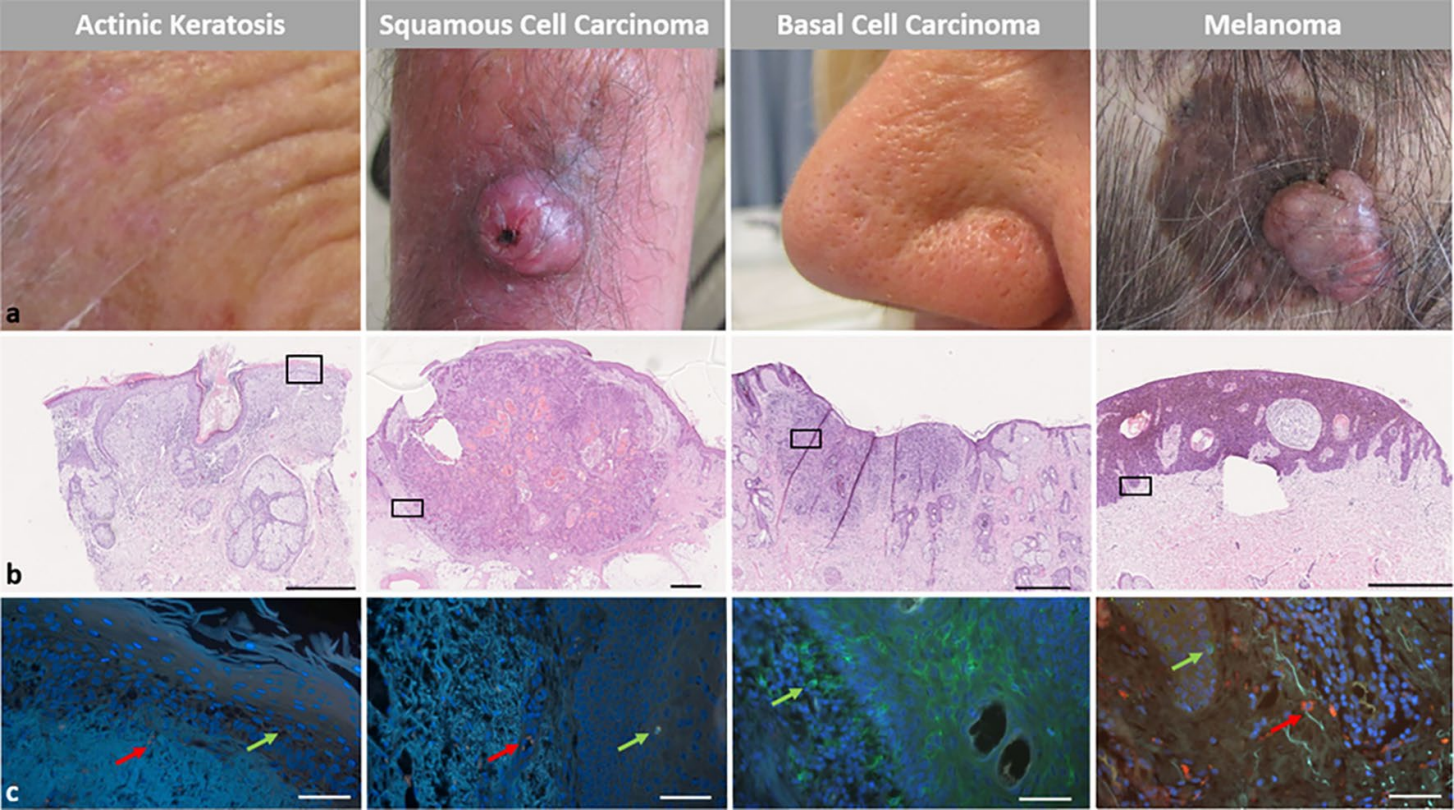
Clinical presentation and histopathological features of skin tumor types. Representative images of four different skin tumors are shown: AK, SCC, BCC, and melanoma. The top panel (a) shows the clinical appearance of each tumor type with characteristic macroscopic features. The middle panel (b) shows corresponding hematoxylin and eosin (H&E)-stained histological sections of each tumor type, highlighting their distinct architectural and cytological features. The bottom panel (c) shows IF staining for CD1a^+^ DC (green) and CD3^+^ T cells (red) in a representative region of interest (as indicated in the H&E staining). Green arrows indicate CD1a^+^ DC, and red arrows indicate CD3^+^ T cells. Scale bars, 1000 μm for histological overview images (500 μm for AK; b), and 50 μm for IF images

Figure 3 presents representative images providing a comprehensive overview of the clinical, histopathological, and immunological characteristics of the analyzed tumor types. The top panel illustrates the clinical presentation of AK, SCC, BCC, and melanoma, showing their distinct morphological features. The middle panel shows hematoxylin and eosin (H&E)-stained sections, revealing tumor-specific architectural and cytological features, such as epidermal dysplasia in AK, keratinocyte atypia in SCC, basaloid nests in BCC, and melanocytic proliferation in melanoma. The bottom panel shows IF staining of CD1a^+^ DC (green) and CD3^+^ T cells (red) in representative tumor sections. Green arrows indicate CD1a^+^ DC, and red arrows indicate CD3^+^ T cells, highlighting their distribution within the TME. These images illustrate the morphological and immunological heterogeneity across tumor entities.

### Localization of CD1a^+^ DC in different types of skin cancer

**Fig. 4:**
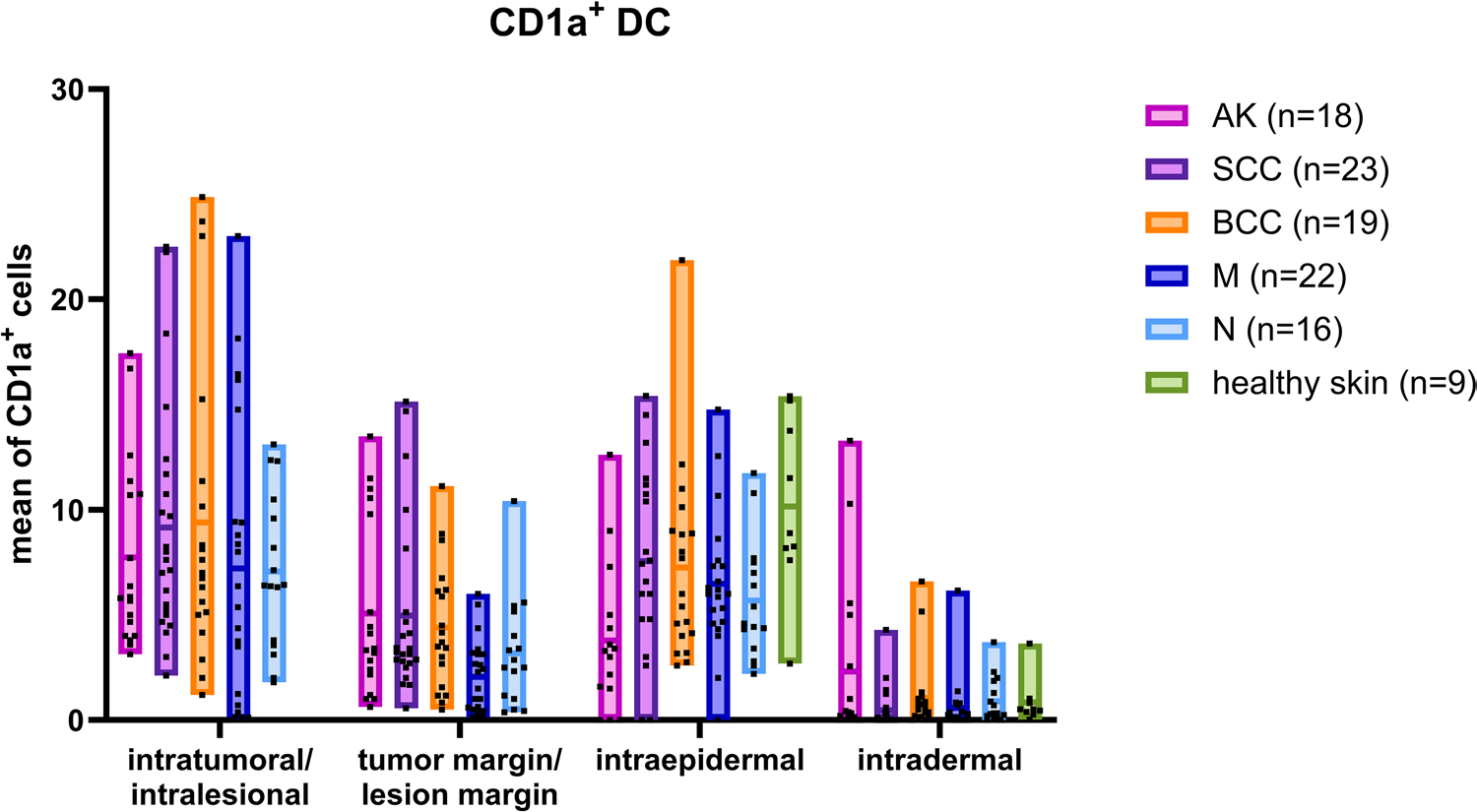
Distribution of CD1a^+^ DC in AK (pink, n = 18), SCC (purple, n = 23), BCC (orange, n = 19), and melanoma (dark blue, n = 22) compared to nevi (light blue, n = 16) and healthy skin (green, n = 9). Data are presented asfl oating bar charts showing the minimum to maximum values, with the mean indicated as the central line. Individualdata points are shown as black squares. Each data point represents one biological sample, calculated as the meanvalue of 5–8 regions of interest (ROIs) per sample. The counts were assessed separately in four regions as defined in Figure 1: intratumoral/intralesional, tumor margin/lesion margin, intraepidermal, and intradermal. Statistical analysiswas performed using MANOVA to assess overall effects, followed by one-way ANOVA for region-specific comparisons and post hoc testing where applicable

CD1a^+^ DC, which play a key role in antitumor immunity by presenting antigens to T cells, were present in all tumor types. MANOVA revealed significant differences among regions (*F*_3,82_ = 70.396, *p* < 0.001) and among entities (Pillai's trace: *F*_12,252_ = 2.771, *p* = 0.001), suggesting tumor entity-specific spatial distribution patterns (Fig. 4).

In AK samples, CD1a^+^ DC were most abundant in the intratumoral area (7.73 ± 4.48 cells/field), with moderate numbers at the tumor margin (5.07 ± 4.20 cells/field) and lower counts in the intraepidermal (3.79 ± 3.54 cells/field) and intradermal regions (2.32 ± 4.00 cells/field).

In SCC samples, the highest number of CD1a^+^ DC was observed in the intratumoral region (9.17 ± 5.68 cells/field), followed by the tumor margin (4.98 ± 4.16 cells/field). The intraepidermal area had a relatively higher density of CD1a^+^ DC compared to AK (7.57 ± 4.53 cells/field), whereas the intradermal area had a notably low number of CD1a^+^ DC (0.47 ± 1.02 cells/field).

BCC showed the highest intratumoral density among all tumor types (9.40 ± 7.22 cells/field), with moderate densities at the tumor margin (4.41 ± 2.99 cells/field) and in the epidermis (7.27 ± 4.61 cells/field). The dermis was sparsely populated (1.06 ± 1.76 cells/field).

In contrast, melanoma samples exhibited a distinct distribution pattern. Similar to other skin cancers, the intratumoral region had the highest concentration of CD1a^+^ DC (7.21 ± 6.80 cells/field). However, the tumor margin had a lower density (2.01 ± 1.75 cells/field) than that observed in NMSC. The intraepidermal region showed a moderate presence of CD1a^+^ DC (6.48 ± 3.27 cells/field), whereas the dermal region contained a very low number (0.55 ± 1.34 cells/field).

In nevi, CD1a^+^ DC were most abundant intralesionally (7.07 ± 3.07 cells/field), as well as in the epidermis (5.69 ± 2.86 cells/field), lesion margin (3.19 ± 2.60 cells/field), and dermis (0.90 ± 1.06 cells/field). Compared to melanoma, nevi exhibited a higher density of DC at the lesion margin, although this difference was not statistically significant (*p* = 0.089, η^2^ = 0.083). Pairwise comparisons between melanoma and nevi revealed no statistically significant differences in CD1a^+^ DC density across the regions investigated (*p* > 0.1).

In healthy skin, CD1a^+^ DC were primarily located in the epidermis, with a mean of 11.53 (± 3.37 cells/field), and only a few were found in the adjacent dermis (0.89 ± 1.08 cells/field). In contrast to the decreased numbers observed across all four tumor entities (AK, SCC, BCC, melanoma), healthy skin showed the highest average number of CD1a^+^ DC in the epidermis.

Overall, all tumor types demonstrated an enrichment of CD1a^+^ DC within the tumor. Healthy skin exhibited the highest intraepidermal density of DC, while melanoma was characterized by a relative scarcity of DC at the tumor margin. This suggests an impairment in immune surveillance in this region. Statistical comparisons confirmed these observations. Although no entity-specific differences were detected in overall or intratumoral (*p* > 0.3) CD1a^+^ DC densities, significant variation emerged at the tumor margin (*F*_3,78_ = 3.68, *p* = 0.015, η^2^ = 0.12) and in the epidermis (*F*_3,70_ = 2.91, *p* = 0.040, η^2^ = 0.11). Post hoc analyses revealed that melanoma harbored significantly fewer CD1a^+^ DC at the tumor margin than BCC and SCC (*p* < 0.05), while AK showed intermediate values. Similarly, intraepidermal CD1a^+^ DC numbers were lower in melanoma than in SCC and BCC. There was a trend toward significance in the dermal compartment (*p* = 0.052), indicating low overall CD1a^+^ DC densities across entities. Effect size estimates indicated that the entity contributed moderately to the variance in tumor margins (η^2^ = 0.12) and the epidermis (η^2^ = 0.11), whereas the intratumoral and dermal compartments showed small effects only (η^2^ ≤ 0.03). No statistically significant differences in CD1a^+^ DC densities were detected when comparing melanoma and nevi across intratumoral/intralesional, intraepidermal, or intradermal compartments (all *p* > 0.3). At the tumor margin/lesion margin, however, there was a non-significant trend toward lower CD1a^+^ DC numbers in melanoma (*p* = 0.089, η^2^ = 0.083), which suggests an early impairment of DC-mediated immune surveillance in malignant versus benign melanocytic lesions. Consistent with these findings, post hoc analyses revealed significantly higher intraepidermal CD1a^+^ DC densities in healthy skin than in all tumor entities (*p* < 0.001).

### Localization of CD3^+^ T cells in different types of skin cancer

**Fig. 5:**
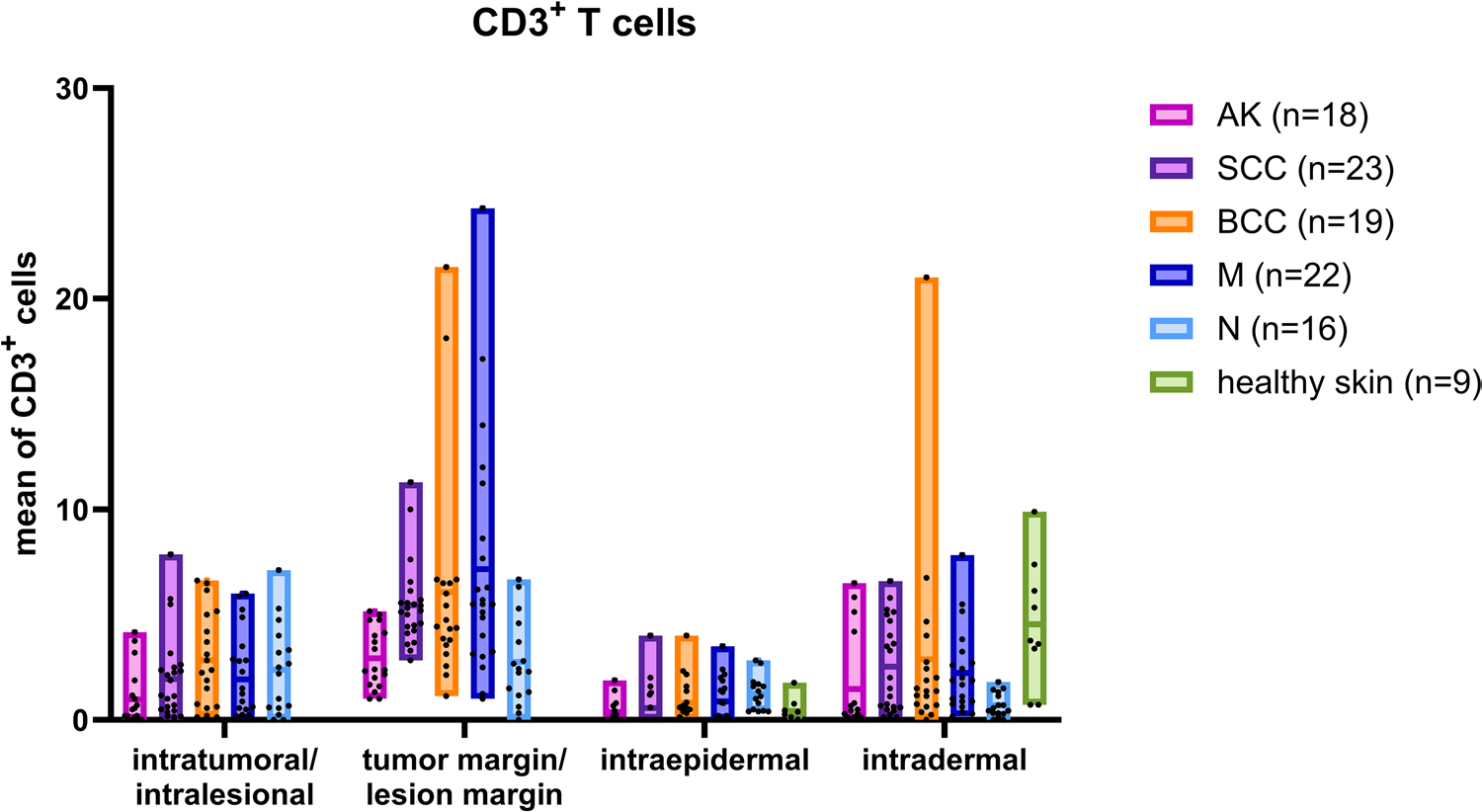
Distribution of CD3^+^ T cells in AK (pink, n = 18), SCC (purple, n = 23), BCC (orange, n = 19), and melanoma (dark blue, n = 22) compared to nevi (light blue, n = 16) and healthy skin (green, n = 9). Data are presented as floating bar charts showing the minimum to maximum values, with the mean as a central line. Individual data points are shown as black squares within the bars. Each data point represents one biological sample, calculated as the mean value of 5–8 regions of interest (ROIs) per sample. The different regions were analyzed separately as shown in Figure 1: intratumoral/intralesional, tumor margin/lesion margin, intraepidermal, and intradermal. Statistical analysis was performed using MANOVA to assess overall effects, followed by one-way ANOVA for region-specific comparisons and post hoc testing where applicable

CD3^+^ T cells, which are crucial for adaptive immune responses, were present in all tumor types and in all regions examined. The highest densities were consistently found at the tumor margin. A MANOVA confirmed a significant main effect of tumor type on CD3^+^ T cell densities (Pillai’s trace: *F*_20,332_ = 2.12, *p* = 0.004), indicating that distribution patterns varied across tumor types (Fig. 5).

In AK samples, the tumor margin exhibited the highest concentration of CD3^+^ T cells (2.93 ± 1.43 cells/field). The dermal region displayed a lower density (1.47 ± 2.30 cells/field), while the intratumoral and intraepidermal regions contained a low number of T cells (0.98 ± 1.36 and 0.38 ± 0.58 cells/field, respectively).

In SCC, CD3^+^ T cells were likewise most abundant at the tumor margin (5.45 ± 1.97 cells/field), with a moderate density in the dermal region (2.53 ± 2.18 cells/field). There were fewer T cells in the intratumoral region (1.94 ± 2.03 cells/field) and almost no T cells in the epidermal layer (0.56 ± 1.05 cells/field). 

The tumor margin of BCC samples exhibited the highest density of CD3^+^ T cells (6.08 ± 5.12 cells/field), followed by the dermal region (2.88 ± 4.70 cells/field). A similar number of T cells was observed in the intratumoral region (2.83 ± 2.25 cells/field). In contrast, the epidermis showed a very sparse T cell infiltration (0.82 ± 1.06 cells/field). 

In melanoma samples, the tumor margin exhibited the greatest concentration of CD3^+^ T cells (7.17 ± 5.60 cells/field), followed by the dermal region (2.23 ± 1.97 cells/field). The intratumoral region showed a comparable T cell density (1.92 ± 2.05 cells/field). As with the other tumor types, the epidermis contained fewer T cells (0.87 ± 1.04 cells/field).

In nevi, CD3^+^ T cells were detected in all regions, with the greatest numbers observed at the lesion margin (2.75 ± 2.06 cells/field) and within the intralesional region (2.38 ± 2.13 cells/field). Lower densities were observed in the epidermis (1.25 ± 0.81 cells/field) and dermis (0.70 ± 0.57 cells/field). Compared to nevi, melanoma samples displayed markedly higher T cell infiltration, particularly at the tumor margin/lesion margin (7.17 ± 5.60 vs. 2.75 ± 2.06 cells/field) and in the dermis (2.23 ± 1.97 vs. 0.70 ± 0.57 cells/field). Intratumoral T cell numbers were slightly lower in melanoma samples (1.92 ± 2.05 vs. 2.38 ± 2.13 cells/field). Epidermal densities were similar (0.87 ± 1.04 vs. 1.25 ± 0.81 cells/field). Direct comparison of melanoma and nevi revealed significantly higher CD3^+^ T cell densities in melanoma at the tumor margin/lesion margin (*F*_1,34_ = 8.52, *p* = 0.006, η^2^ = 0.20) and in the dermis (*F*_1,34_ = 8.06, *p* = 0.008, η^2^ = 0.19). However, intratumoral/intralesional and epidermal densities were not significantly different (*p* = 0.43 and *p* = 0.26).

In healthy skin, CD3^+^ T cells were rarely found in the epidermis (0.39 ± 0.56 cells/field), whereas most T cells were located in the dermis (4.50 ± 3.00 cells/field).

Across entities, ANOVA confirmed highly significant differences at the tumor margin (*F*_5,100_ = 111.25, *p* < 0.001, η^2^ ≈ 0.85). Here, melanoma harbored significantly more CD3^+^ T cells than AK, SCC, and BCC (all *p* < 0.01). Nevi showed intermediate values. Dermal infiltration also varied (*F*_5,97_ = 3.21, *p* = 0.01, η^2^ = 0.14), with SCC and BCC showing higher values than melanoma and AK. No significant variation was observed intratumorally (*p* = 0.18) or in the epidermis (*p* = 0.12).

### Correlation between CD1a^+^ DC and CD3^+^ T cells

Correlation analyses revealed a weak but statistically significant positive association between CD1a^+^ DC and CD3^+^ T cell densities in the intratumoral region (Spearman’s *ρ* = 0.27, *p* = 0.005) and at the tumor margin (*ρ* = 0.26, *p* = 0.007). In contrast, no significant correlations were observed in the epidermal (*p* = 0.29) or dermal (*p* = 0.32–0.51) regions. These findings suggest that the spatial coupling of DC and T cell infiltration primarily occurs within the tumor core and at the invasive front, rather than in the surrounding tissue layers.

### Sex- and age-related differences in immune cell infiltration

No significant sex-related differences were observed in the overall densities of CD1a^+^ DC or CD3^+^ T cells. Independent-samples* t* tests confirmed no significant variation in total CD1a^+^ DC (20.15 ± 12.76 vs. 18.53 ± 11.67, *p* = 0.55) or CD3^+^ T cells (10.94 ± 8.74 vs. 9.75 ± 5.50, *p* = 0.73) between female and male patients.

Region-wise analyses likewise showed no sex-related effects for either cell type (CD1a^+^ DC: intratumoral *p* = 0.82, tumor margin *p* = 0.93, intraepidermal *p* = 0.55, intradermal *p* = 0.71; CD3^+^ T cells: intratumoral *p* = 0.19, tumor margin *p* = 0.27, intraepidermal *p* = 0.62, intradermal *p* = 0.30, Supplementary Table 10). Entity-specific analyses confirmed this overall pattern: AK, SCC, and BCC showed no sex-dependent differences in either cell type (all *p* > 0.2), except for a significant difference in the epidermis of SCC, where male patients displayed slightly higher CD3^+^ counts (*p* = 0.029). In melanoma, there were no significant differences in CD1a^+^ DC densities between sexes (all *p* > 0.1), though a non-significant intratumoral increase was seen in male patients (9.6 vs. 4.8 cells/field, *p* = 0.10), while CD3^+^ T cell densities remained unaffected (all *p* > 0.25).

A non-significant trend was observed for total CD3^+^ T cell densities (*p* = 0.054). Further confirmation of the absence of sex-related effects on CD1a^+^ DC or CD3^+^ T cell densities was provided by correlation analyses (Pearson’s and Spearman’s; all *r* < 0.12, all *p* > 0.18).

Age-related analysis (< 65 vs. ≥ 65 years) revealed no differences in CD1a^+^ DC densities overall (*p* = 0.98) or by region (all *p* > 0.1). Patients aged ≥ 65 years had significantly higher dermal CD3^+^ T cell counts (2.9 vs. 1.4 cells/field, *p* = 0.02), with a non-significant trend toward higher numbers at the tumor margin (*p* = 0.19). No differences were observed in the intratumoral or intraepidermal regions. When analyzed across all regions combined, CD3^+^ T cells were also more abundant in older patients (11.5 vs. 8.5 cells/field, *p* = 0.05, *d* = 0.43) (Supplementary Table 11). Entity-specific analyses confirmed this pattern. In AK, older patients showed significantly higher CD3^+^ T cell densities (7.8 ± 4.8 vs. 3.8 ± 2.8 cells/field, *p* = 0.040), with no effect on CD1a^+^ DC (*p* = 0.77). SCC showed a similar, albeit non-significant trend (11.3 vs. 8.0 cells/field, *p* = 0.075), while BCC demonstrated numerically higher CD3^+^ T cell counts in older patients (15.4 vs. 10.6 cells/field, *p* = 0.32), with no change in CD1a^+^ DC (*p* = 0.81). There were no age-related differences in melanoma for either cell population (all *p* > 0.6). Correlation analyses using age as a continuous variable confirmed these findings. Although there was no significant association between patient age and CD1a^+^ DC densities (all *p* > 0.1), there was a positive correlation between CD3^+^ T cell densities and age, particularly in the dermis (Spearman’s *ρ* = 0.30, *p* = 0.007) and across all regions combined (*ρ* = 0.27, *p* = 0.013). For nevi, age-related analyses were not feasible, as all patients were under 63 years of age. For healthy skin samples, stratification by sex or age was not possible, as these data were anonymized and therefore unavailable.

### Comparison of IF and immunohistochemistry data

**Fig. 6:**
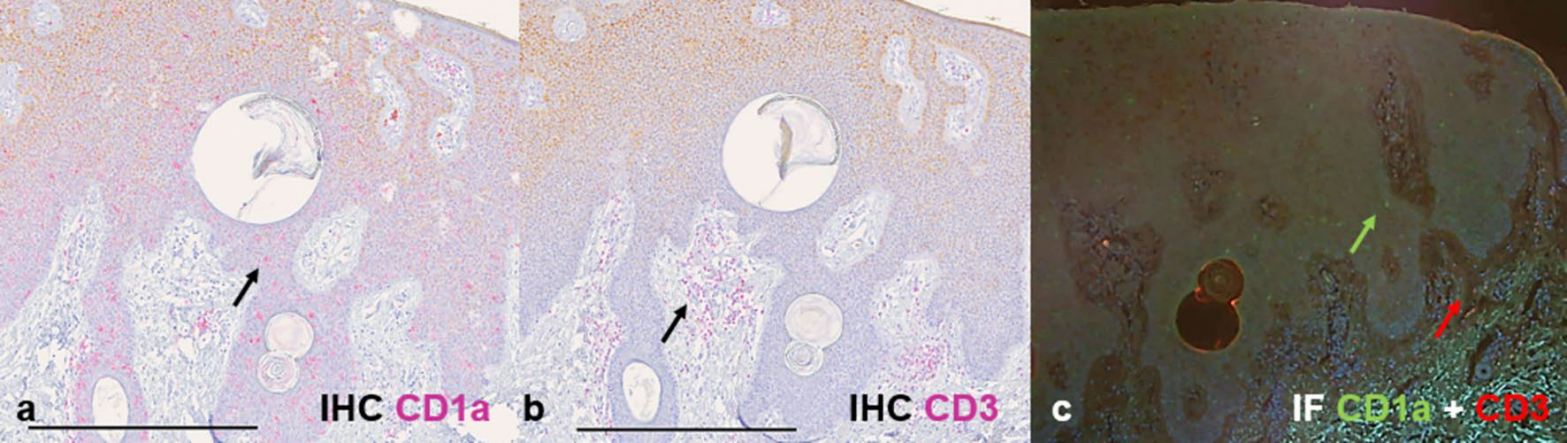
Comparison of IF and IHC staining methods in a melanoma tissue specimen. Representative images comparing the immunostaining patterns of CD1a and CD3 by IHC (a: CD1a^+^ DC in pink, b: CD3^+^ cells in pink, with arrows indicating representative cells), and by IF (c: CD1a^+^ DC in green and CD3+ cells in red, arrows indicate representative cells). Scale bars: 500 μm for IHC images (a and b)

Figure 6 shows a comparison of the IF and immunohistochemistry (IHC) staining methods, focusing on a representative melanoma sample. This comparison highlights the consistency between the two techniques in detecting CD1a^+^ DC and CD3^+^ T cells within the TME. In the IHC sections, CD1a^+^ DC cells (Fig. 6a) cells  and CD3^+^ (Fig. 6b) are stained pink, with arrows indicating representative cells ^+^ . In the IF section (Fig. 6c), the CD1a^+^ DC are stained green (green arrow) and the CD3^+^ T cells are stained red (red arrow).

**Fig. 7:**
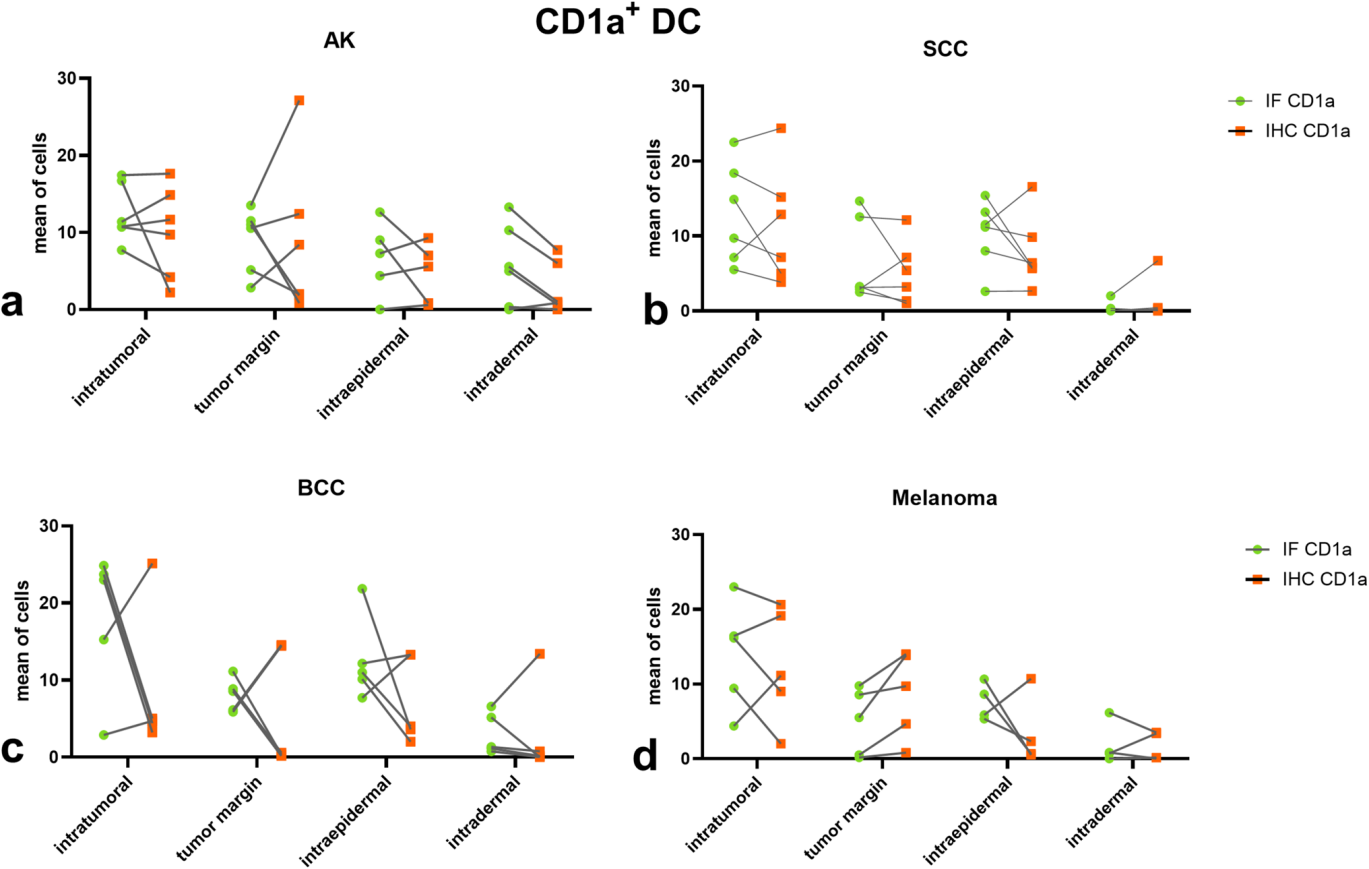
Comparison of CD1a⁺ DC distribution across different types of skin cancer using IF and IHC. The distribution of CD1a⁺ DC across four predefined tissue compartments (intratumoral, tumor margin, intraepidermal, and intradermal) is shown for actinic keratosis (AK; n = 6, a), squamous cell carcinoma (SCC; n = 6,b), basal cell carcinoma (BCC; n = 5, c), and melanoma (n = 5, d). IF (green circles) and IHC (orange squares) were performed on serial sections of the same tumor specimens, enabling direct paired comparison. Individual paired measurements are connected by lines

**Fig. 8:**
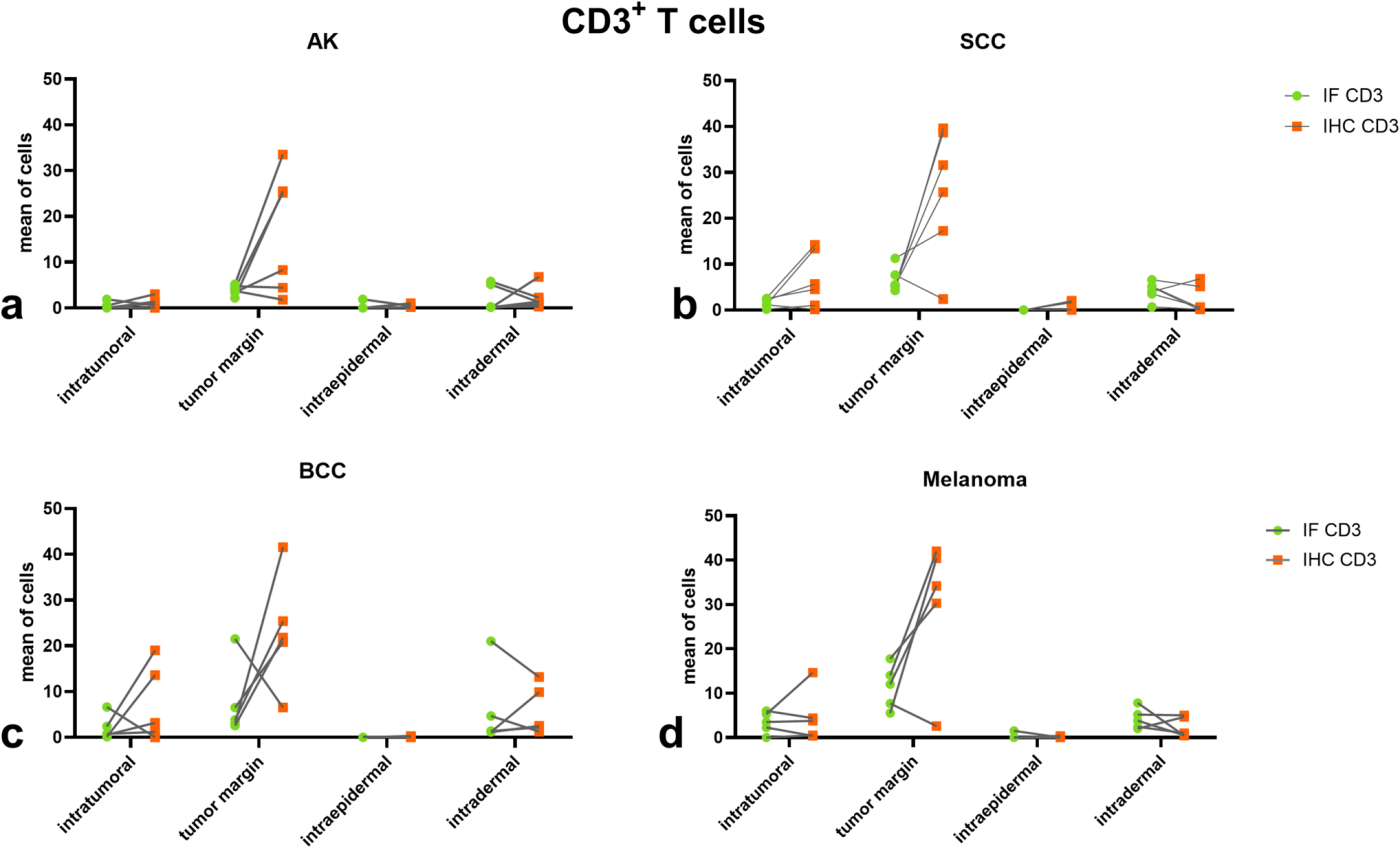
Comparison of CD3⁺ T cell distribution across different types of skin cancer using IF and IHC. The distribution of CD3⁺ T cells across four predefined tissue compartments (intratumoral, tumor margin, intraepidermal, and intradermal) is shown for actinic keratosis (AK; n = 6, a), squamous cell carcinoma (SCC; n = 6, b), basal cell carcinoma (BCC; n = 5, c), and melanoma (n = 5, d). IF (green circles) and IHC (orange squares) were performed on serial sections of the same tumor specimens, enabling direct paired comparison. Individual paired measurements are connected by lines

To compare IF and IHC across tumor entities, mixed-effects models were applied (Supplementary Table 11). For CD1a^+^ DC, no significant effect of staining method was detected in any tumor type, and no significant region × method interactions were observed, indicating comparable quantification between IF and IHC (Fig. 7). For CD3^+^ T cells, no significant method effect was observed in AK, BCC, or melanoma. However, SCC showed a significant main effect (*p* = 0.0245), with higher counts detected by IHC. Region × method interactions were identified in SCC, BCC, and melanoma, indicating compartment-dependent variability (Fig. 8). Overall, IF and IHC demonstrated largely comparable quantitative results across tumor entities, with limited marker- and region-specific differences (Supplementary Table 12).

## Discussion

This study provides valuable insights into the spatial distribution of CD1a^+^ DC and CD3^+^ T cells across different types of skin cancer, demonstrating entity- and region-specific immune landscapes. Using a retrospective design and IF-based spatial mapping of the intratumoral/intralesional, tumor margin/lesion margin, intraepidermal, and intradermal areas, we achieved a high-resolution characterization of immune cell localization.

CD1a^+^ DC were predominantly localized intratumorally and intraepidermally, showing a region-specific distribution.

A key observation was the higher number of CD1a^+^ DC at the tumor margin in NMSC compared to melanoma. An increased density of DC at the tumor margin in BCC has been associated with higher numbers of myofibroblasts and smaller tumor size (Rybka et al. 2008). High densities of conventional myeloid DC subsets (cDC1, cDC2) correlate with improved disease-free survival (Plesca et al. 2020).

Compared to NMSC and healthy skin, melanoma was distinct in showing markedly fewer CD1a^+^ DC at the tumor margin and reduced densities in the overlying epidermis. This may reflect an immune evasion strategy driven by the heterogeneity of the TME (Sikorski et al. 2025; Sosa Cuevas et al. 2023). This finding aligns with previous studies reporting a decrease in epidermal DC overlying primary melanoma lesions (Dyduch et al. 2017). Reduced mature DC density and impaired DC function, together with MHC class I loss, have been associated with poor prognosis (Ladányi et al. 2007). Furthermore, suppression of migratory DC subsets by melanoma cells has been associated with local recurrence and metastasis (van den Hout et al. 2017; Mestrallet et al. 2022).

Healthy skin, by contrast, showed the highest epidermal CD1a^+^ DC densities, consistent with prior reports that tumor-associated skin exhibits a marked depletion of DC compared to non-tumorous controls (Pogorzelska-Dyrbuś and Szepietowski 2020; de Jong and Ogg 2021; Haniffa et al. 2015).

CD3^+^ T cells, the main adaptive effector population, were present in all tumors, but exhibited a distinct distribution pattern compared to CD1a^+^ DC (Fig. 5). As shown in the results, multivariate analysis confirmed a significant main effect of region, with T cells consistently accumulating at the tumor margin.

Another notable finding was the variation in the number of CD3^+^ T cells in tumor tissue and at the tumor margin in AK, SCC, BCC, and melanoma, with melanoma showing the highest numbers, followed by SCC, then BCC, and the lowest in AK.

The consistent accumulation of CD3^+^ T cells at the tumor margin is a hallmark of an active immune barrier at the invasive front, particularly in melanoma. This immune phenotype is characterized by robust T cell infiltration and is classified as a “hot tumor” (Mousa et al. 2024; Sikorski et al. 2025; Bida et al. 2024). This immunologically inflamed phenotype is associated with a high tumor mutational burden and increased neoantigen load, which facilitates T cell recruitment and activation (Sang et al. 2024). Tumor-infiltrating lymphocytes (TIL), including CD3^+^, CD4^+^, CD8^+^, FOXP3^+^, and CD20^+^ cells, have been shown to play a favorable prognostic role in the overall survival of patients with melanoma (Fu et al. 2019; Vargas et al. 2024). Furthermore, patients with a high density of T cells in both the intratumoral and tumor margin regions have been found to have longer survival times and lower recurrence rates (Plesca et al. 2020). In fact, a tumor that is immunologically “inflamed” with high T cell infiltration is generally associated with better disease control, whereas T cell-poor “cold” tumors tend to metastasize or relapse more rapidly (de Oliveira et al. 2024; Sang et al. 2024). The presence, phenotype, and localization of TILs are key determinants of antitumor immunity and therapeutic response, particularly to ICIs (Maibach et al. 2020; Prokopi et al. 2021; Plesca et al. 2020; Sang et al. 2024; van Duin et al. 2024).

PD-1 blockade has been shown to effectively reactivate exhausted, antigen-experienced CD8^+^ T cells in patients with melanoma (Huang et al. 2017). Although melanoma typically exhibits reduced DC numbers at the tumor margin, recent evidence suggests that restoring DC function is critical for maximizing the efficacy of ICI (Prokopi et al. 2021). Recent single-cell data suggest that even sparse, mature DC subsets can sustain T cell activation and predict favorable outcomes (Yang et al. 2025). Responders to anti-PD-1 therapy typically show higher baseline and expanding CD8^+^ T cells at the tumor margin, correlating with tumor regression (Gobbini et al. 2025). Thus, melanoma’s responsiveness to PD-1 inhibition reflects the reactivation of a highly functional, antigen-experienced T cell population within an immunogenic microenvironment rather than the absolute density of DC or T cells.

Compared to nevi, melanoma showed a non-significant trend toward lower levels of CD1a^+^ DC at the tumor or lesion margin (*p* = 0.089), suggesting early impairment of DC-mediated immune surveillance during malignant transformation (Dyduch et al. 2017; Vermi et al. 2003). Melanoma harbored significantly more T cells at the tumor margin and in the dermis, while no differences were observed intratumorally/intralesionally or intraepidermally, suggesting that altered T cell recruitment may serve as an early indicator of malignant transformation in melanocytic lesions.

When considering the healthy control skin, the mean number of dermal CD3^+^ cells across all four tumor entities was lower (2.23 ± 0.60 cells/field) than in the control group (4.5 ± 3.00 cells/field), indicating alterations in immune cell dynamics within the TME.

The spatial interplay between DC and T cells is crucial for effective antitumor immunity, as DC act as professional antigen-presenting cells and orchestrate T cell activation. In our study, correlation analyses revealed a weak but significant positive association between CD1a^+^ DC and CD3^+^ T cells in the intratumoral region and at the tumor margin. In contrast, no significant correlations were observed in the epidermal or dermal regions. These findings imply that spatial coupling of DC and T cell infiltration primarily occurs within the tumor core and at the invasive front, whereas the surrounding tissue layers appear to follow independent regulatory mechanisms. Recent studies showed that the formation of cDC1–CD8^+^ T cell clusters, especially in the tumor margin, is associated with increased T cell activation, proliferation, and cytotoxicity, and correlates with clinical response to immune checkpoint blockade (Gobbini et al. 2025).

Although we found no significant sex-related differences in the densities of CD1a^+^ DC or CD3^+^ T cells across tumor types, previous studies have suggested that sex can influence systemic immunity and clinical outcomes. Women generally mount stronger immune responses, which may impact cancer progression and treatment efficacy (Wang et al. 2019). Large national cohort studies have shown that women treated with ICIs demonstrate significantly better overall and progression-free survival than men (Petersen et al. 2024). These disparities have been attributed to hormonal influences and sex-related differences in immune system function, including variations in T cell activity and cytokine profiles, which may modulate ICI efficacy (Xiao et al. 2024; Shi et al. 2021). Overall, our data suggest that these clinical disparities are not reflected by baseline densities of CD1a^+^ DC or CD3^+^ T cells in the TME, indicating that functional differences rather than cell numbers may underlie the observed effects.

Age emerged as a more relevant determinant of immune infiltration than sex. Older patients (≥ 65 years) exhibited significantly higher intradermal CD3^+^ T cell counts, with an increased accumulation at the tumor margin. However, the intratumoral and epidermal regions remained unaffected. This pattern was most evident in AK, with similar but non-significant trends in SCC and BCC, consistent with the concept of “inflammaging,” whereby chronic inflammation and cumulative UV damage promote T cell recruitment to keratinocyte-derived tumors (Lee et al. 2021; Salminen et al. 2022; Berman and Cockerell 2013). However, it remains unclear whether these infiltrates reflect functional antitumor immunity or exhausted populations. In contrast, melanoma showed no age-related differences, suggesting that its strong intrinsic immunogenicity and immune evasion mechanisms may override demographic influences (Kalaora et al. 2022).

Nevi largely resembled melanoma, but showed a trend toward higher densities of CD1a^+^ DC at the margin, while healthy skin exhibited the highest densities of CD1a^+^ DC in the epidermis. Due to limited or anonymized data, sex- and age-related analyses were not feasible in these groups, which represents a limitation of the study. The sample size per entity was limited (*n* = 16–23). Moreover, only CD1a and CD3 were analyzed, without functional or phenotypic subtyping (e.g., cDC1/2, CD4/CD8), which restricts conclusions regarding immune quality. Despite these limitations, our study provides valuable insights into the number and localization of CD1a^+^ DC and CD3^+^ T cells in different types of skin cancer.

The use of IF enabled the precise spatial mapping of CD1a^+^ DC and CD3^+^ T cells in tumor tissues, providing a complementary approach to IHC. Overall, quantitative results were largely comparable between the two methods, with no significant method-dependent differences for CD1a^+^ dendritic cells. For CD3^+^ T cells, however, region-dependent differences were detected in SCC, suggesting compartment-specific variability between techniques. A possible explanation lies in the detection of densely packed immune cell aggregates. In areas with pronounced lymphocytic infiltration, especially at the tumor margin, chromogenic IHC staining may lead to partial signal overlap, making it more difficult to distinguish closely adjacent CD3^+^ cells. In contrast, IF enables clearer optical separation of signals and may facilitate the identification of individual cells within dense clusters. Taken together, both techniques are suitable for quantitative immune profiling. However, IF may offer advantages in resolving cellular aggregates and preserving spatial detail, particularly in highly inflamed tumor regions.

## Conclusion

Our study demonstrates region-specific infiltration patterns of CD1a^+^ DC and CD3^+^ T cells across major types of skin cancer. CD1a^+^ DC predominated within intratumoral and intraepidermal regions, whereas T cells were primarily enriched at the tumor margin and in the intradermal compartment. These spatial differences indicate selective, region-specific immune interactions within the TME. Compared with NMSC, melanoma displayed markedly fewer CD1a^+^ DC at the tumor margin but stronger T cell infiltration, suggesting divergent mechanisms of immune regulation and escape. Age, but not sex, influenced T cell infiltration, particularly in keratinocyte-derived tumors, consistent with inflammation-driven immune recruitment in aging skin. The weak yet significant spatial association between CD1a^+^ DC and CD3^+^ T cells emphasizes the importance of local DC–T cell dynamics at the tumor–immune interface. Overall, our findings highlight that antitumor immunity in skin cancer is region-dependent. Further studies involving larger and phenotypically defined immune cell populations are needed to fully explore the functional implications of these spatial immune patterns and their potential relevance for predicting therapeutic response.

## Supplementary Information

Below is the link to the electronic supplementary material.

Supplementary file1 (DOCX 31 KB)

## Data Availability

The datasets generated and analyzed during the current study are available from the corresponding author upon reasonable request.

## Abbreviations

AK: Actinic keratosis
BCC: Basal cell carcinoma
BSA: Bovine serum albumin
CD3: Cluster of differentiation 3
cDC1: Conventional type 1 dendritic cell
cDC2: Conventional type 2 dendritic cell
DAPI: 4′,6-diamidino-2-phenylindole
DC: Dendritic cell
FFPE: Formalin-fixed, paraffin-embedded
H&E: Hematoxylin and eosin
ICIs: Immune checkpoint inhibitors
IHC: Immunohistochemistry
IF: Immunofluorescence
LC: Langerhans cells
MANOVA: Multivariate analysis of variance
MHC class I: Major histocompatibility complex class I
MHC class II: Major histocompatibility complex class II
NMSC: Non-melanoma skin cancer
PD-1: Programmed cell death protein 1
PBS: Phosphate-buffered saline
RT: Room temperature
SCC: Squamous cell carcinoma
SPSS: Statistical Package for the Social Sciences
TILs: Tumor-infiltrating lymphocytes
TME: Tumor microenvironment
UV: Ultraviolet

## Declarations

### Editorial assistance

Artificial intelligence (ChatGPT-4o, OpenAI, San Francisco, CA, USA) was used for linguistic optimization, grammar correction, and structural editing without altering the scientific content.
