# Supplementary material for: Localization of dendritic cells and T cells within the tumor microenvironment in different types of skin cancer
Source: Histochem Cell Biol. 2026 Apr 25;164(1):28. doi: 10.1007/s00418-026-02468-8 (PMC13110200; doi:10.1007/s00418-026-02468-8)
Supplement: Supplementary file 1 — Supplementary file1 (DOCX 31 KB) [file 418_2026_2468_MOESM1_ESM.docx]

**Supplementary - Localization of Dendritic Cells and T Cells within the Tumor Microenvironment in Different Types of Skin Cancer**

Marina Wanner^1#^, Anna Brunner^1^, Daniela Ortner-Tobider^1^, Christoph H Tripp^1^, Martin Hermann^2^, Selina Neurauter^3^, Barbara Del-Frari^3^, Van Anh Nguyen^1*^, Patrizia Stoitzner^1*^

1 Department of Dermatology, Venereology, and Allergology, Medical University of Innsbruck, Innsbruck, Austria

2 Department of Anesthesia and Intensive Care Medicine, Medical University Innsbruck, Innsbruck, Austria

3 Department of Plastic, Reconstructive and Aesthetic Surgery, Medical University Innsbruck, Innsbruck, Austria

* contributed equally

# Marina Wanner, University Clinic of Dermatology, Venereology, and Allergology, Innsbruck, Anichstraße 35, 6020 Innsbruck, Austria.[marina.wanner@i-med.ac.at](mailto:marina.wanner@i-med.ac.at), https://orcid.org/0000-0001-6930-6997

*Supplementary Table 1. Demographic data of the study group (n= 82).*

| Sample, ***n*** |  | **40** |
| --- | --- | --- |
| Age, ***in years*** | Mean ± SD  Range | 65.9 (± 12.01)  33-92 |
| Sex, ***n (%)*** | Female  Male | 36 (43.9)  46 (56.1) |
| Entity, ***n (%)*** | Actinic keratosis  Squamous cell carcinoma  Basal cell carcinoma  Melanoma | 18 (22)  23 (28)  19 (23.2)  22 (26.8) |
| Localization, ***n (%)*** | Head/ Neck  Upper extremity  Lower extremity  Trunk | 40 (48.8)  11 (13.4)  13 (15.9)  18 (22.0) |

Supplementary Table 2. Demographic data of actinic keratosis (n= 18).

| Actinic keratosis | **Sample**, *n* |  | **18** |
| --- | --- | --- | --- |
|  | **Age**, *in years* | Mean ± SD | 64.17 (± 6.09) |
|  | **Sex**, *n (%)* | Female  Male | 3 (16.7)  15 (83.3) |
|  | **Localization**, *n (%)* | Head/ Neck  Upper extremity  Lower extremity  Trunk | 16 (88.9)  /  /  2 (11.1) |

*Supplementary Table 3. Demographic data of squamous cell carcinoma (n=23).*

| Squamous cell carcinoma | **Sample**, *n* |  | **23** |
| --- | --- | --- | --- |
|  | **Age**, *in years* | Mean ± SD | 69.4 (±12.9) |
|  | **Sex**, *n (%)* | Female  Male | 9 (39.1)  14 (60.9) |
|  | **Localization**, *n (%)* | Head/ Neck  Upper extremity  Lower extremity  Trunk | 9 (39.1)  4 (17.4)  5 (21.7)  5 (21.7) |
|  | **Type**, *n (%)* | \| Bowen's disease \| \| --- \| \| Bowen's carcinoma \| \| Keratoacanthoma \| \| invasive SCC \| | \| 4 (17.4) \| \| --- \| \| 3 (12.5) \| \| 3 (12.5) \| \| 13 (56.5) \| |

*Supplementary Table 4. Demographic data of basal cell carcinoma (n=19).*

| Basal cell carcinoma | **Sample**, *n* |  | **19** |
| --- | --- | --- | --- |
|  | **Age**, *in years* | Mean ± SD | 59.8 (±10.5) |
|  | **Sex**, *n (%)* | Female  Male | 13 (68.4)  6 (31.6) |
|  | **Localization**, *n (%)* | Head/ Neck  Upper extremity  Lower extremity  Trunk | 9 (47.4)  2 (10.5)  2 (10.5)  6 (31.6) |
|  | **Types** *n (%)* | \| Superficial BCC \| 3 \| \| --- \| --- \| \| Nodular BCC \| 2 \| \| Sclerodermiform BCC \| 2 \| \| Other BCC \| 1 \| | \| 7 (36.8) \| \| --- \| \| 6 (31.6) \| \| 5 (26.3) \| \| 1 (5.3) \| |

*Supplementary Table 5. Demographic data of melanoma (n=22).*

| Melanoma | **Sample**, *n* |  | **22** |
| --- | --- | --- | --- |
|  | **Age**, *in years* | Mean ± SD | 69.00 (±14.2) |
|  | **Sex**, *n (%)* | Female  Male | 11 (50)  11 (50) |
|  | **Localization**, *n (%)* | Head/ Neck  Upper extremity  Lower extremity  Trunk | 6 (27.3)  5 (22.7)  6 (27.3)  5 (22.7) |
|  | **Breslow thickness, n (%)** | T1 > 1.0 mm  T2 1.01‑2.0 mm  T3 2.01‑4.0 mm  T4 > 4.1 mm | 2 (9.1)  5 (22.7)  5 (22.7)  10 (45.5) |

*Supplementary Table 6. Demographic data of nevus (n=16).*

| Nevus | **Sample**, *n* |  | **16** |
| --- | --- | --- | --- |
|  | **Age**, *in years* | Mean ± SD | 38.13 (± 13.01) |
|  | **Sex**, *n (%)* | Female  Male | 6 (37.5)  10 (62.5) |
|  | **Localization**, *n (%)* | Head/ Neck  Upper extremity  Lower extremity  Trunk | 1 (6.3)  2 (12.5)  /  13 (81.3) |

Supplementary Table 7. Mean (± SD)CD1a expression (per field of view) for each tumor entity within the study group.

|  | **AK** | **SCC** | **BCC** | **M** |
| --- | --- | --- | --- | --- |
| intratumoral,  *Mean of cells (± SD)* | 7.73  (± 4.48) | 9.17  (± 5.68) | 9.40  (± 7.22) | 7.21  (± 6.80) |
| tumor margin,  *Mean of cells (± SD)* | 5.07  (± 4.20) | 4.98  (± 4.16) | 4.41  (± 2.99) | 2.01  (± 1.75) |
| epidermal,  *Mean of cells (± SD)* | 3.79  (± 3.54) | 7.57  (± 4.53) | 7.27  (± 4.61) | 6.48  (± 3.27) |
| dermal,  *Mean of cells (± SD)* | 2.32  (± 4.00) | 0.47  (± 1.02) | 1.06  (± 1.76) | 0.55  (± 1.34) |
|  |  |  |  |  |

Supplementary Table 8. Mean (± SD) CD3 expression (per field of view) for each tumor entity within the study group.

|  | **AK** | **SCC** | **BCC** | **M** |
| --- | --- | --- | --- | --- |
| intratumoral,  *Mean of cells (± SD)* | 0.98  (± 1.36) | 1.94  (± 2.03) | 2.83  (± 2.25) | 1.92  (± 2.05) |
| tumor margin,  *Mean of cells (± SD)* | 2.93  (± 1.43) | 5.45  (± 1.97) | 6.08  (± 5.12) | 7.17  (± 5.60) |
| epidermal,  *Mean of cells (± SD)* | 0.38  (± 0.58) | 0.56  (± 1.05) | 0.82  (± 1.06) | 0.87  (± 1.04) |
| intradermal,  *Mean of cells (± SD)* | 1.47  (± 2.30) | 2.53  (± 2.18) | 2.88  (± 4.70) | 2.23  (± 1.97) |

Supplementary Table 9. Mean numbers (± SD) of CD1a⁺ dendritic cells and CD3⁺ T cells per high-power field in control samples (nevi and healthy skin). Data are shown separately for four regions (intratumoral, tumor margin, epidermal, intradermal).

|  | **Nevus CD1a** | **Healthy skin CD1a** | **Nevus CD3** | **Healthy skin CD3** |
| --- | --- | --- | --- | --- |
| intratumoral,  *Mean of cells (± SD)* | 7.07  (± 3.07) | / | 2.38  (± 2.13) | / |
| tumor margin,  *Mean of cells (± SD)* | 3.19  (± 2.60) | / | 2.75  (± 2.06) | / |
| epidermal,  *Mean of cells (± SD)* | 5.69  (± 2.86) | 11.53  (± 3.37) | 1.25  (± 0.81) | 0.39  (± 0.56) |
| intradermal,  *Mean of cells (± SD)* | 0.90  (± 1.06) | 0.89  (± 1.08) | 0.70  (± 0.57) | 4.5  (± 3.00) |

*Supplemental Table 10. Sex-related differences in CD1a⁺ dendritic cells and CD3⁺ T cells in tumor samples*

| Region | **Marker** | **Female (Mean ± SD)** | **Male (Mean ± SD)** | **p-value** | **Cohen’s d** |
| --- | --- | --- | --- | --- | --- |
| All regions | CD1a | 20.15 ± 12.76 | 18.53 ± 11.67 | 0.55 | 0.13 |
| Intratumoral | CD1a | 8.31 ± 6.94 | 8.43 ± 5.47 | 0.82 | 0.05 |
| Tumor margin | CD1a | 4.25 ± 3.75 | 3.95 ± 3.47 | 0.93 | –0.02 |
| Epidermal | CD1a | 7.12 ± 4.76 | 5.82 ± 3.61 | 0.55 | \| –0.30 \| \| --- \| |
| Dermal | CD1a | 0.89 ± 1.63 | 1.14 ± 2.72 | 0.71 | \| 0.08 \| \| --- \| |
| All regions | CD3 | 10.94 ± 8.736 | 9.75 ± 5.53 | 0.73 | –0.05 |
| Intratumoral | CD3 | 1.76 ± 1.90 | 2.07 ± 2.13 | 0.19 | \| –0.15 \| \| --- \| |
| Tumor margin | CD3 | 6.08 ± 5.15 | 5.06 ± 3.23 | 0.27 | –0.24 |
| Epidermal | CD3 | 0.48 ± 0.83 | 0.85 ± 1.06 | 0.62 | \| –0.11 \| \| --- \| |
| Dermal | CD3 | 2.72 ± 3.75 | 1.98 ± 2.11 | 0.30 | 0.24 |

*Supplemental Table 11. Age-related differences (<65 vs. ≥65 years) in CD1a⁺ dendritic cells and CD3⁺ T cells in tumor samples*

| Region | **Marker** | **<65 (Mean ± SD)** | **≥65 (Mean ± SD)** | | **p-value** | **Cohen’s d** |
| --- | --- | --- | --- | --- | --- | --- |
| All regions | CD1a | 19.28 ± 13.96 | | 19.22 ± 10.77 | 0.98 | 0.01 |
| Intratumoral | CD1a | 8.77 ± 6.55 | | 8.11 ± 5.85 | 0.63 | 0.11 |
| Tumor margin | CD1a | 4.50 ± 4.10 | | 3.79 ± 3.15 | 0.38 | 0.20 |
| Epidermal | CD1a | 5.56 ± 4.58 | | 7.00 ± 3.85 | 0.15 | –0.34 |
| Dermal | CD1a | 1.19 ± 2.18 | | 0.92 ± 2.38 | 0.62 | 0.11 |
| All regions | CD3 | 8.52 ± 6.42 | | 11.52 ± 7.37 | 0.05 | –0.43 |
| Intratumoral | CD3 | 1.83 ± 2.19 | | 2.01 ± 1.92 | 0.70 | 0.09 |
| Tumor margin | CD3 | 4.78 ± 4.25 | | 6.02 ± 4.15 | 0.19 | –0.30 |
| Epidermal | CD3 | 0.64 ± 0.93 | | 0.70 ± 1.01 | 0.79 | –0.06 |
| Dermal | CD3 | 1.43 ± 1.75 | | 2.90 ± 3.44 | 0.02 | –0.51 |

*Supplemental Table 12. Summary of mixed-effects model analyses comparing IF and IHC across tumor entities.*

| Tumor entity | Marker | | n | | Region p | | Method p | | Method x region p | |
| --- | --- | --- | --- | --- | --- | --- | --- | --- | --- | --- |
| AK | | | CD1a | 6 | 0.0084 | | 0.37 | | 0.81 | |
| AK | | | CD3 | 6 | 0.0178 | | 0.0748 | | 0.0644 | |
| SCC | | | CD1a | 6 | 0.0002 | | 0.4712 | | 0.2544 | |
| SCC | | | CD3 | 6 | <0,0001 | | **0.0245** | | 0.0012 | |
| BCC | | | CD1a | 5 | 0.017 | | 0.3386 | | 0.0974 | |
| BCC | | | CD3 | 5 | 0.0007 | | 0.1635 | | 0.0372 | |
| Melanoma | | | CD1a | 5 | 0.0008 | | 0.7004 | | 0.1406 | |
| Melanoma | | | CD3 | 5 | <0.0001 | | 0.0980 | | 0.0085 | |
